# Genetic Characterization of Chikungunya Virus in Field-Caught *Aedes aegypti* Mosquitoes Collected during the Recent Outbreaks in 2019, Thailand

**DOI:** 10.3390/pathogens8030121

**Published:** 2019-08-02

**Authors:** Proawpilart Intayot, Atchara Phumee, Rungfar Boonserm, Sriwatapron Sor-suwan, Rome Buathong, Supaporn Wacharapluesadee, Narisa Brownell, Yong Poovorawan, Padet Siriyasatien

**Affiliations:** 1 Medical Science Program, Faculty of Medicine, Chulalongkorn University, Bangkok 10330, Thailand; 2 Thai Red Cross Emerging Infectious Diseases-Health Science Centre, World Health Organization Collaborating Centre for Research and Training on Viral Zoonoses, Chulalongkorn Hospital, Bangkok 10330, Thailand; 3 Vector Biology and Vector Borne Disease Research Unit, Department of Parasitology, Faculty of Medicine, Chulalongkorn University, Bangkok 10330, Thailand; 4 Bureau of Epidemiology, Department of Disease Control, Ministry of Public Health, Nonthaburi 11000, Thailand; 5 Center of Excellence in Clinical Virology, Faculty of Medicine, Chulalongkorn University, Bangkok 10330, Thailand

**Keywords:** genetic characterization, Chikungunya virus, *Aedes aegypti*, Thailand

## Abstract

Chikungunya virus (CHIKV) is a mosquito-borne virus belonging to the genus *Alphavirus*. The virus is transmitted to humans by the bite of infected female *Aedes* mosquitoes, primarily *Aedes aegypti*. CHIKV infection is spreading worldwide, and it periodically sparks new outbreaks. There are no specific drugs or effective vaccines against CHIKV. The interruption of pathogen transmission by mosquito control provides the only effective approach to the control of CHIKV infection. Many studies have shown that CHIKV can be transmitted among the *Ae. aegypti* through vertical transmission. The previous chikungunya fever outbreaks in Thailand during 2008–2009 were caused by CHIKV, the East/Central/South African (ECSA) genotype. Recently, there have been 3794 chikungunya cases in 27 provinces reported by the Bureau of Epidemiology of Health Ministry, Thailand during 1 January–16 June 2019; however, the cause of the re-emergence of CHIKV outbreaks is uncertain. Therefore, the aims of this study were to detect and analyze the genetic diversity of CHIKV infection in field-caught mosquitoes. Both female and male *Ae. aegypti* were collected from endemic areas of Thailand, and CHIKV detection was done by using *E1*-nested RT-PCR and sequencing analysis. A total of 1646 *Ae. aegypti* samples (900 females and 746 males) were tested. CHIKV was detected in 54 (3.28%) and 14 samples (0.85%) in female and male mosquitoes, respectively. Seventeen samples of female *Ae. aegypti* collected from the Ubon Ratchathani, Chiang Rai, Chiang Mai, Nakhon Sawan, and Songkhla provinces found mutation at *E1*: A226V. Interestingly, *E1*: K211E mutation was observed in 50 samples collected from Nong Khai, Bangkok, Prachuap Khiri Khan, and Krabi. In addition, the phylogenetic tree indicated that CHIKV in *Ae. aegypti* samples were from the Indian Ocean Clade and East/South African Clade. Both clades belong to the ECSA genotype. The information obtained from this study could be used for prediction, epidemiological study, prevention, and effective vector control of CHIKV. For instance, a novel CHIKV strain found in new areas has the potential to lead to a new outbreak. Health authorities could plan and apply control strategies more effectively given the tools provided by this research.

## 1. Introduction

Chikungunya virus (CHIKV) is a species of the genus *Alphavirus* belonging to the family *Togaviridae*, which has a genome consisting of a linear, positive-sense, single-stranded RNA molecule of approximately 11.8 kb. The genome of CHIKV is considered to be 5′ cap-nsP1-nsP2-nsP3-nsP4-(junction region)-C-E3-E2-6K-E1-poly (A) 3′ [1,2]. CHIKV consists of three genotypes: East/Central/South African (ECSA), West African (WA), and Asian. Vazeille et al. (2007) revealed that the ECSA genotype carrying a new variant of mutation at alanine-to-valine (A226V) in the *E1* protein has spread in areas where *Aedes albopictus* (Skuse) is predominant. This mutation has shown to be increasing the fitness of the virus for *Ae. albopictus* mosquitoes as well as its infectivity [3].

CHIKV infection is one of the emerging and re-emerging mosquito-borne viral diseases transmitted by *Aedes* mosquitoes such as *Ae. aegypti* and *Ae. albopictus*, the Asian tiger mosquito associated with several recent large-scale epidemics [4,5]. CHIKV was initially transmitted through the vector *Ae. aegypti*, whereas *Ae. albopictus* showed a mutation in the *E1*-A226V of CHIKV for improving transmissibility to humans [6,7,8]. CHIKV infection has become a serious public health problem in many parts of the world. CHIKV was first identified in Tanzania in 1952 [9], and an outbreak occurred in a large tropical area (i.e., Africa, South Asia, and Southeast Asia) [10]. Moreover, cases of CHIKV from Europe, the USA, and Australia through travelers returning from affected areas were also recently reported [11,12]. In Thailand, the first reported case of CHIKV infection in Bangkok caused by the Asian genotype occurred in 1958 [13]. In 2008, a CHIKV epidemic was reported as the ECSA genotype [14,15,16].

CHIKV infection is characterized by high-grade fever, nausea, rash, and severe arthralgia. The symptoms are generally of short duration (i.e., approximately between 2–12 days), although some patients may have recurrent episodes for several weeks after infection [17,18,19]. CHIKV also involves systemic infection in many organ systems (e.g., renal, hepatic, cardiac, neurologic, and respiratory) [20]. There are no specific drugs or CHIKV-specific vaccines against the disease. Supportive treatment is mainly used to relieve the symptoms. CHIKV is increasing public health concerns due to its prevalence, geographical distribution, and severity; however, the options to help control these infections are still limited. Therefore, vector control is the principle strategy used to control CHIKV outbreaks.

The transovarial transmission in mosquitoes is one of mechanisms of CHIKV’s maintenance in nature during adverse climatic conditions. During this period, the infected mosquito becomes inactive or unable to survive, and therefore the virus can establish a persistent infection within the mosquito. The virus disseminates to infect the ovary of the female mosquito and then infects the egg during oviposition [21]. Importantly, the eggs of *Aedes* mosquitoes are generally resistant to desiccation and are able to survive for a long period of time, leading to the persistence of CHIKV in their eggs. Therefore, the virus can persist through the larva stage and continue to the adult stage [22]. Moreover, previous studies reported that CHIKV transmission involves several *Aedes* species. For instance, *Ae. aegypti* has been identified as the main vector of CHIKV, and the transovarial transmission of the virus has been reported [23]. However, the number of mosquito generations through which the virus has successfully persisted has not been identified. Recently, it was found that CHIKV (Indian Ocean Lineage) isolated from *Ae. albopictus* in Southern Thailand could be transmitted vertically under laboratory conditions up to F5 and F6 generations of *Ae. aegypti* and *Ae. albopictus*, respectively [24].

*Ae. albopictus* has also been reported as a potential vector in Asia [5]. There are reports of vectors of CHIKV in Africa including *Ae. africanus* in East Africa; *Ae. cordellieri* and *Ae. taylori* in South Africa; and *Ae. furcifer, Ae. dalzielii, Ae. luteocephalus, Ae. taylori,* and *Ae. vittatus* in West Africa [25,26]. In Thailand, the Bureau of Epidemiology of Thailand Health Ministry reported 3794 of chikungunya cases in 27 provinces from 1 January–16 June 2019 [27]. However, the re-emergence of CHIKV outbreaks in Thailand is unpredictable, and effective disease control relies on vector mosquito control measures only. It is necessary to understand the transmission cycle of CHIKV associated with mosquito vectors in endemic areas. Therefore, in this study, we focused on the detection of CHIKV infection in both female and male *Ae. aegypti* around patients’ homes in different regions of Thailand by using *E1*-nested RT-PCR. The data could be valuable for designing effective therapeutic strategies, vector control measures, and eradication of CHIKV associated with *Ae. aegypti* mosquitoes in Thailand.

## 2. Results

### 2.1. Molecular Detection of CHIKV RNA in Ae. aegypti

**Figure 1:**
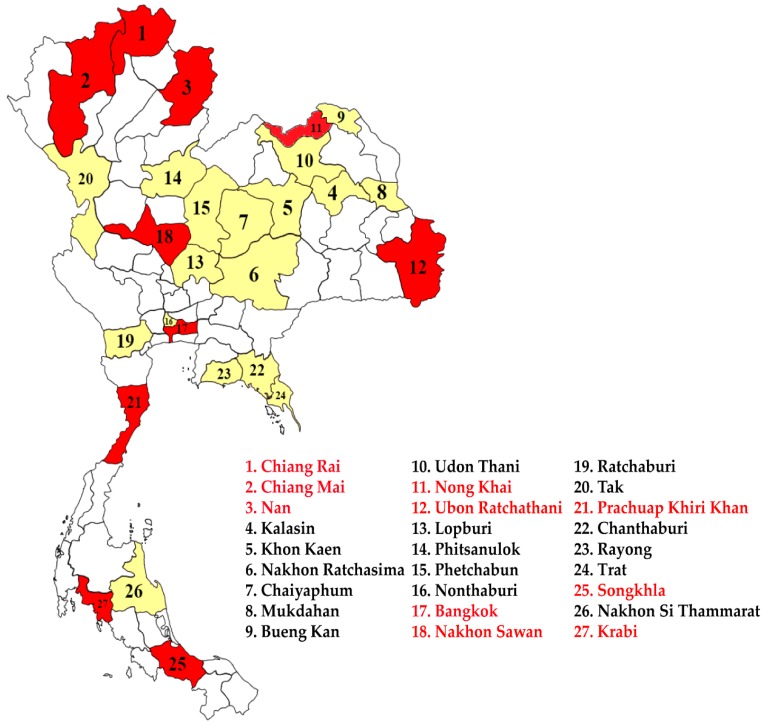
Map of Thailand showing locations of the sample collection sites in the 27 provinces of 6 regions in affected areas. Red indicates the collection locations of positive CHIKV in mosquito samples and yellow indicates negative CHIKV in mosquito samples.

In this study, we developed new primers for the specific detection of the mutation at alanine-to-valine in the *E1* glycoprotein at position 226 (*E1*: A226V) in infected mosquitoes. The total samples used for CHIKV detection consisted of 900/1646 female and 746/1646 male *Ae. aegypti* mosquitoes, which were collected from the endemic area of CHIKV infection. Most samples were found around patients’ homes from 27 provinces of 6 regions of Thailand (Figure 1). Our findings showed that CHIKV RNA were positive in 3.28% and 0.85% in female and male *Ae. aegypti* mosquitoes from Northern (Chiang Rai, Chiang Mai, and Nan), Northeastern (Nong Khai and Ubon Ratchathani), Central (Bangkok and Nakhon Sawan), and Southern (Songkhla, Prachuap Khiri Khan, and Krabi) regions, respectively, whereas CHIKV was not detected in *Ae. aegypti* samples from the Eastern region using *E1*-nested RT-PCR (Table 1).

### 2.2. Sequencing and Phylogenetic Analysis

**Figure 2:**
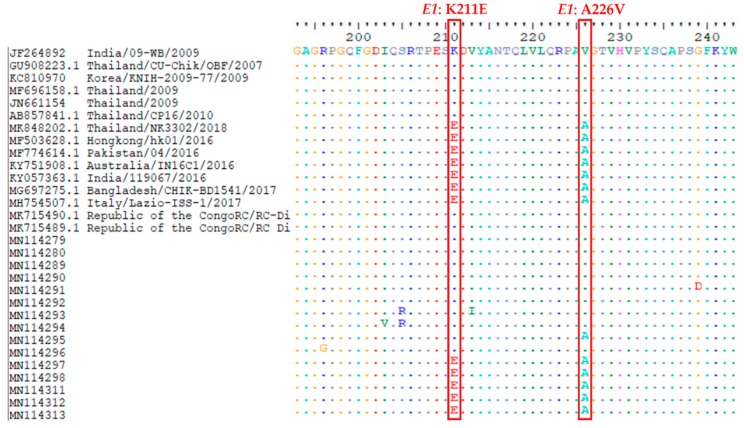
Showing a portion of the alignment of amino acid sequences of the *E1* gene of CHIKV in *Ae. aegypti* at positions of the A226V and K211E mutations, indicated by red vertical columns.

**Figure 3:**
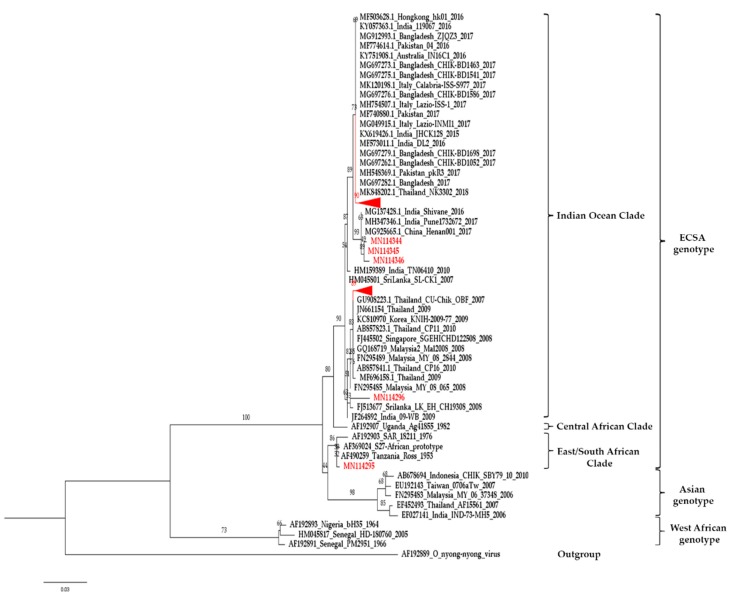
Phylogenetic tree of CHIKV in mosquitoes constructed from partial *E1* sequences from all regions of Thailand. The maximum likelihood was constructed with IQ-TREE by using the maximum-likelihood method with 1000 ultrafast bootstrap replicates. The best-fit model of substitution was found using the auto function on the IQ-TREE web server. The sequences from this study are indicated with a red color. Code of MN114344, MN114345, and MN114346 is CHIKV-*Ae. aegypti* from Nong Khai, and MN114296 and MN114295 are CHIKV-*Ae. aegypti* from Songkhla and Nan, respectively.

A total of 68 sequences of *E1*-CHIKV from *Ae. aegypti* were obtained from five geographical regions of Thailand. Most of the sequences contained 539 bp. The BLAST results showed that the sequence was also similar to CHIKV isolated from the plasma and serum of patients in Malaysia (Accession no. KT324228) and India (Accession no. MK473628 and MK473637) with >99% identity and a full 100% coverage of the nucleotide sequences. The genetic variations of CHIKV from *Ae. aegypti* collected from different geographic regions showed a maximum of 4.3% between CHIKV-*Ae. aegypti* from Prachuap Khiri Khan and Nan provinces. The intraspecific variation analysis in each province showed 0–1.9% variation. Interestingly, we found 17 (1.03%) of CHIKV from female of *Ae. aegypti* collected from Ubon Ratchathani (10 samples); 6 samples from Chiang Rai, Chiang Mai, and Nakhon Sawan (two samples from each province); and 1 sample from Songkhla province. All sequences from this outbreak revealed that the *E1*: A226V mutation was similar to the sequences isolated from the serum of patients in the previous outbreak from Thailand, Malaysia, Korea, Singapore, India, and Sri Lanka during 2008–2010. Additionally, the substitution of lysine by glutamic acid at position 211 (*E1*: K211E) was observed in all 50 samples collected from Nong Khai (3 samples), Bangkok (14 samples), Prachuap Khiri Khan (32 samples), and Krabi (1 sample). The sequences of *E1*: K211E were similar to CHIKV isolated from the outbreak in Bangladesh, Pakistan, India, Hong Kong, Australia, and Italy during 2016–2017 and in Thailand 2018 (Figure 2, Figure S1). The phylogenetic tree indicates that the CHIKV in *Ae. aegypti* samples could be clearly classified into two clades consisting of Indian Ocean Clade and East/South African Clade. Both clades belong to the ECSA genotype. One sample of CHIKV in *Ae. aegypti* from Nan province was of the East/South African Clade (Figure 3). All sequences of CHIKV in *Ae. aegypti* were submitted to the GenBank database under accession numbers MN114279-MN114346.

## 3. Discussion

Chikungunya fever is an emerging arbovirus infection, representing a silent worldwide health problem. In Thailand, the previous outbreak of CHIKV in 1958 was of the Asian genotype [13]. However, the CHIKV outbreak in late 2008 was of the ECSA genotype [14,15,16], which was 99–100% closely related to CHIKV from the outbreaks in India in 2007 [28] and Singapore in 2008 [29]. In Thailand during 2008–2009, the large outbreak of CHIKV (ECSA genotype) in the southern provinces also rapidly spread across Thailand, with more than 50,000 infected cases with the *E1*: A226V mutation [30]. In 2013, Wanlapakorn et al. reported an outbreak of CHIKV infection in the northeastern region, Bueng Kan province, and suggested that these isolations in the Indian Ocean clade contained the *E1*: A226V mutation and were grouped into the ECSA genotype. This evidence revealed the presence and continued circulation of the *E1*: A226V mutation in the Indian Ocean clade of CHIKV in Thailand since 2008 [31]. Recently, the re-emergence of CHIKV was reported by the Bureau of Epidemiology of Thailand Health Ministry, as mentioned previously [27]. However, little information is available about the molecular epidemiology of CHIKV transmitted from the mosquitoes, *Ae. Aegypti*, in Thailand. In the present study, we focused on the genetic divergence of sequence data of CHIKV from both female and male *Ae. aegypti* collected from endemic areas of Thailand. The results showed that CHIKV RNA was detected in female *Ae. aegypti* (3.28%) more than male *Ae. aegypti* (0.85%). A previous report showed the infection rate of CHIKV in both female and male pools of *Ae. aegypti* (8.5%) and *Ae. albopictus* (7.6%) collected from the southern part of Thailand [5]. Additionally, CHIKV RNA were only detected in 6.4% of *Ae. aegypti* (3.8% females and 2.6% larvae) from Eastern Thailand [32]. In Vietnam, CHIKV-positive individual female *Ae. aegypti* were detected in two (0.18%) samples from Dac Nong and Long An [33]. In the literature, it is suggested that *Ae. aegypti* might be a vector of CHIKV and also could support the vertical transmission of CHIKV in *Ae. aegypti*. Several reports detected transovarial transmission of ECSA genotype CHIKV under natural conditions in field-caught *Ae. aegypti* and *Ae. albopictus* in different parts of the world [34,35,36]; moreover, under laboratory conditions, the transovarial transmission has also been demonstrated in both *Ae. aegypti* and *Ae. albopictus* [23,24]. Honório et al. (2019) has recently suggested the first vertical transmission of Asian genotype CHIKV in Brazilian and Florida populations of *Ae. albopictus* in the laboratory [37]. However, in this study, we only collected *Ae. aegypti* because we wanted to focus on studying inside patients’ houses, in which *Ae. aegypti* are commonly found. These mosquitoes usually rest inside the houses after feeding on human blood. Therefore, complete studies on *Ae. albopictus* and CHIKV in Thailand should be done in the future.

It is possible to demonstrate the co-circulation of both Asian and ECSA genotypes in Thailand. Therefore, the present study demonstrates the genetic diversity of CHIKV in field-caught *Ae. aegypti* mosquitoes collected from endemic areas of Thailand. Our results show that 17 samples found the *E1*: A226V mutation from female of *Ae. aegypti*; moreover, we found that the *E1*: K211E mutation was observed in 50 samples from both female and male *Ae. aegypti* collected from Nong Khai, Bangkok, Prachuap Khiri Khan, and Krabi. For phylogenetic analyses, molecular evolution analyses of *E1* indicated that all positive CHIKV samples were classified into Indian Ocean and East/South African clades. Both clades were present in the ECSA genotype. We assumed that the widespread and renewed chikungunya fever epidemic in the current outbreak in Thailand may contribute to the molecular evolutionary adaptation acquired by CHIKV as a result of the A226V and K211E mutations in the *E1* gene in *Ae. aegypti* in the different regions of Thailand. In the past, a major epidemic of CHIKV started in many Indian Ocean island nations in early 2005 [38]. In 2007, the implications of A226V mutation have been increased severity, reported mortality, and a large epidemic in Kerala State, India; additionally, *Ae. albopictus* was a potential vector for CHIKV during these outbreaks [39]. Tsetsarkin et al. (2007) revealed that the *E1*: A226V mutation enhances the ability of the salivary gland of *Ae. albopictus* to become infected with a virus, and therefore, increases the capability of the mosquitoes to transmit the virus to another host ; in contrast, the *E1*: A226V mutation has a slightly negative effect on CHIKV infectivity, a negligible effect on dissemination, and a slight positive effect on the transmissibility of CHIKV by *Ae. aegypti* [40]. However, our results showed that all 17 sequences representative from this outbreak revealed *E1*: A226V, as observed in 2008–2010 isolates from the serum of Thai patients as Accession nos. MF696158, AB857823, AB857841, and JN661154, respectively [41]. The appearance and continued circulation of the *E1*: A226V mutation in Thailand has been suspected since 2008. Interestingly, we found the *E1*: K211E mutation in 50 samples of *Ae. aegypti*, which were reported in Bangladesh, Pakistan, India, Hong Kong, Australia, and Italy during 2016–2017 [42]. This evidence supports that CHIKV may have been distributing and evolving in different geographic regions of the world and may have been depending on the prevalence of the vectors and their efficiency in transmitting of CHIKV. Moreover, it might possibly occur through the migration, travel, or residence of people in high-risk areas of CHIKV infection. There is one report showing that *E1*: K211E mutation was detected earlier in the Asian lineage [43], and has been found in the ECSA lineage in one sample from Andhra Pradesh, India [44]. The *E1*: K211E variations have also been invested in the autochthonous imported case in Southeastern France from India in 2010 [45]. Agarwal et al. (2016) showed that the CHIKV in *Ae. aegypti* demonstrated that the *E1*: K211E mutation led to increased infectivity, dissemination, and transmission, whereas the aforementioned mutation was not observed in *Ae. albopictus* [46]. It is interesting to point out that the mutants of *E1*: A226V and *E1*: K211E have been found in both humans and other mosquitoes in Thailand. If these mutated strains occur in a new setting, major CHIKV outbreaks can be predicted.

## 4. Materials and Methods

### 4.1. Ethics Statement

The study was approved by the animal research ethics committee of Chulalongkorn University Animal Care and Use Protocol (CU-ACUP), Faculty of Medicine, Chulalongkorn University, Bangkok, Thailand (COA No. 023/2560).

### 4.2. Sample Collection

A total of 1646 adult mosquitoes were collected in and around the house of confirmed CHIKV-infected patients using mosquito aspirators. The larvae were also collected using a plastic dropper or plastic cup. The collections were conducted in different geographical regions of Thailand, including the Northern (Chiang Rai, Chiang Mai, and Nan), Northeastern (Kalasin, Khon Kaen, Nakhon Ratchasima, Chaiyaphum, Mukdahan, Bueng Kan, Udon Thani, Nong Khai, and Ubon Ratchathani), Central (Lop Buri, Phitsanulok, Phetchabun, Nonthaburi, Bangkok, and Nakhon Sawan), Eastern (Chanthaburi, Rayong, and Trat), Western (Ratchaburi, Tak, and Prachuap Khiri Khan), and Southern (Songkhla, Nakhon Si Thammarat, and Krabi) regions. The mosquito samples were kept in liquid nitrogen and transferred to the laboratory of Vector Biology and Vector-Borne Disease Research Unit, Department of Parasitology, Faculty of Medicine, Chulalongkorn University. Based on their morphological characteristics, all samples were differentiated according to their sex and species. Additionally, the larvae were reared into adult mosquitoes for species identification and then individual mosquitoes were tested for the viral detection.

### 4.3. Viral RNA Extraction

Viral RNA was extracted from individual mosquitoes as follows. First, the mosquito was ground in 1X phosphate-buffered saline (PBS) and centrifuged at 11,000× *g* for 5 min. The supernatant was processed for viral RNA extraction using a viral RNA extraction kit, Invisorb^®^ Spin Virus RNA Mini kit (STRATEC Molecular GmbH, Berlin, Germany), according to the manufacturer’s instructions. The RNA concentration was quantified by a Nano Drop 2000c spectrophotometer (Thermo Fisher Scientific, MA, USA). The extracted RNA samples were used immediately for CHIKV detection, and the samples were maintained for long-term storage at −80 °C.

### 4.4. CHIKV RNA Detection

The RNA extracted from individual mosquitoes were amplified and tested for CHIKV detection using nested RT-PCR. The first amplification was performed using the two outer primer pairs, targeting the *E1* gene of CHIKV (E1-10145 F: 5’-ACAAAACCGTCATCCCGTCTC-3’ genome position 10145-10165 and E1-11158R: 5’-TGACTATGTGGTCCTTCGGAGG-3’ genome position 11137-11158) [47]. The primers used for the second amplification were newly designed based on the *E1* gene sequences, as forward primer 5′-GCGCCTACTGCTTCTGCGA-3′ and reverse primer sequences were 5′-CTTCATCGCTC TTACCGGGT-3′. The first round of PCR reaction was carried out in a final volume of 25 μL using the Superscript III one-step RT-PCR kit (Invitrogen, Grand Island, NY, USA), which consisted of 12.5 μL of 2XR reaction mix, 0.7 μL (10 μM) of each primer, 1 μL of *Taq* polymerase, 4.1 μL of sterile nuclease-free water, and 6.0 μL of RNA template. The PCR cycling conditions were performed with an initial incubation at 50 °C for 30 min, denaturation at 95 °C for 15 min, and followed by 40 cycles of 95 °C for 1 min, 64 °C for 1 min, 72 °C for 1 min, and the final extension at 72 °C for 10 min. Two microliters from the first amplification were further amplified with the inner pairs of the primer in a final volume of 25 μL, containing 2.5 μL of 10X buffer, 2.5 μL (25 mM) of MgCl_2_, 2.5 μL (10 μM) of dNTP (GeneAll, Seoul, Korea), 0.4 μL (10 μM) of each inner primer, 0.2 μL (5U/μL) of *Taq* polymerase (Thermo Fisher Scientific, Waltham, MA, USA), and 14.5 μL of sterile nuclease-free water. The reaction mixture was amplified using the following parameters: 95 °C for 3 min, followed by 40 cycles of 95 °C for 30 s, 62 °C for 30 s, 72 °C for 1 min, and the final step at 72 °C for 7 min. The amplified products were analyzed on a 1.5% agarose gel, stained with ethidium bromide, and then visualized under ultraviolet light with Quantity One Quantification Analysis Software version 4.5.2 (Gel DocEQ System; Bio-Rad, Hercules, CA, USA).

### 4.5. DNA Cloning and Sequencing

The positive PCR amplicons were directly ligated to pGEM-T Easy Vector (Promega, Madison, WI, USA), using T4 DNA ligase, following the manufacturer’s recommendations. The resulting constructs were transformed into competent bacterial cells (*Escherichia coli* DH5α strain) and then the bacterial transformants were screened using the blue–white colony system. The colonies suspected of carrying the DNA fragment were cultured, and the plasmid DNA was isolated using the Invisorb^®^ Spin Plasmid Mini Kit (STRATEC molecular GmbH, Berlin, Germany), according to the manufacturer’s instructions. The purified plasmids were sent to a commercial company (Macrogen, Seoul, Korea) for direct DNA sequencing.

### 4.6. Phylogenetic Tree Construction

The nucleotide sequences were aligned using the BioEdit Sequence Alignment Editor Version 7.2.5. The phylogenetic trees were constructed using the maximum-likelihood method with IQ-TREE on the IQ-TREE web server (http://iqtree.cibiv.univie.ac.at/) assessed by the ultrafast bootstrap with 1000 replicates. The best-fit model of substitution was found using the auto function on the IQ-TREE web server. Eventually, the phylogenetic tree was visualized and edited using FigTree v.1.4.4 software.

## 5. Conclusions

In this study, we highlight the natural infection of CHIKV RNA in female and male *Ae. aegypti* from the chikungunya fever endemic area of Thailand. Moreover, CHIKV-positive samples demonstrated the presence of vertical transmission in the field population of *Ae. aegypti*. The genetic characteristics of CHIKV based on the *E1* sequences showed the ECSA genotype and the mutation of virus containing *E1*: A226V and *E1*: K211E, which were associated with increased infectivity, dissemination, and transmission in the *Ae. aegypti* vector. Widespread surveys throughout the country covering a large area and geographic distribution should be performed for a better understanding of the epidemiology of CHIKV through virus and vertical transmission. The data of molecular evidence provide the basis for studying the epidemiology, prevention, and effective control for CHIKV vector, as well as the chikungunya fever outbreak prediction in Thailand.

## Figures and Tables

**Table 1:** Chikungunya virus (CHIKV) detection in *Aedes aegypti* collected from various regions of Thailand.

Regions	Provinces	Total Sample (n)	Chikungunya-Positive (n/total)	
			Female	Male
Northern	Chiang Rai	50	2/28	0/22
	Chiang Mai	64	2/37	0/27
	Nan	40	1/22	0/18
Northeastern	Kalasin	7	0/5	0/2
	Khon Kaen	63	0/33	0/30
	Nakhon Ratchasima	19	0/12	0/7
	Chaiyaphum	40	0/25	0/15
	Mukdahan	8	0/4	0/4
	Bueng Kan	10	0/7	0/3
	Udon Thani	60	0/32	0/28
	Nong Khai	258	0/145	3/113
	Ubon Ratchathani	75	10/42	0/33
Central	Lopburi	60	0/32	0/28
	Phitsanulok	51	0/27	0/24
	Phetchabun	51	0/32	0/19
	Nonthaburi	21	0/13	0/8
	Bangkok	89	11/51	3/38
	Nakhon Sawan	64	2/35	0/29
Western	Ratchaburi	85	0/47	0/38
	Tak	96	0/52	0/44
	Prachuap Khiri Khan	93	24/46	8/47
Eastern	Chanthaburi	60	0/29	0/31
	Rayong	45	0/22	0/23
	Trat	60	0/29	0/31
Southern	Songkhla	80	1/38	0/42
	Nakhon Si Thammarat	62	0/28	0/34
	Krabi	35	1/27	0/8
Total		1646	54/900	14/746

## Supplementary Materials

The following are available online at https://www.mdpi.com/2076-0817/8/3/121/s1, Figure S1: Showing full of alignment of amino acid sequences of the *E1* gene of CHIKV in *Ae. aegypti* at positions of the A226V and K211E mutations.
