# Supplementary figures and images for: Genetic Characterization of Chikungunya Virus in Field-Caught Aedes aegypti Mosquitoes Collected during the Recent Outbreaks in 2019, Thailand
Source: Pathogens. 2019 Aug 2;8(3):121. doi: 10.3390/pathogens8030121 (PMC6789480; doi:10.3390/pathogens8030121)

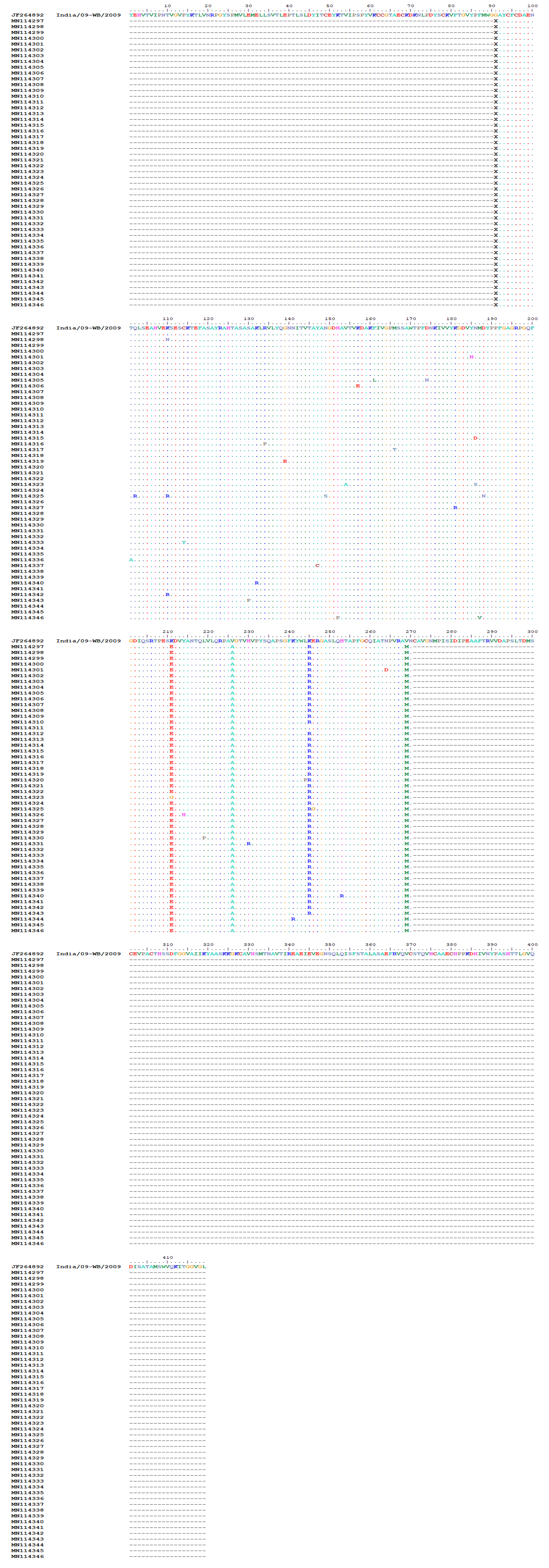

Supplement: Supplementary file 1 [file pathogens-08-00121-s001.zip › Figure. S1.png]
